# Measurement challenges in shared decision making: putting the ‘patient’ in patient‐reported measures

**DOI:** 10.1111/hex.12380

**Published:** 2015-06-25

**Authors:** Paul J. Barr, Glyn Elwyn

**Affiliations:** ^1^ The Dartmouth Institute for Health Policy and Clinical PracticeDartmouth CollegeHanoverNHUSA; ^2^ The Dartmouth Center for Health Care Delivery ScienceDartmouth CollegeHanoverNHUSA

**Keywords:** decision making, patient participation, physician–patient relations, psychometrics/instrumentation

## Abstract

Measuring clinicians' shared decision‐making (SDM) performance is a key requirement given the intensity of policy interest in many developed countries – yet it remains one of the most difficult methodological challenges, which is a concern for many stakeholders. In this Viewpoint Article, we investigate the development of existing patient‐reported measures (PRMs) of SDM identified in a recent review. We find that patients were involved in the development of only four of the 13 measures. This lack of patient involvement in PRM development is associated with two major threats to content validity, common to all 13 PRMs of SDM: (i) an assumption of patient awareness of ‘decision points’ and (ii) an assumption that there is only one decision point in each healthcare consultation. We provide detailed examples of these threats and their impact on accurate assessment of SDM processes and outcomes, which may hamper efforts to introduce incentives for SDM implementation. We propose cognitive interviewing as a recommended method of involving patients in the design of PRMs in the field of SDM and provide a practical example of this approach.

## Shared decision making – a measurement challenge

The methodological challenge of measuring shared decision making (SDM) is increasingly relevant to health professionals and policymakers.^1^, ^2^, ^3^, ^4^ In the USA, the quality of patient‐centred care, including SDM, may soon be incentivized with financial remuneration for health professionals,^3^, ^5^ and in the UK, the inclusion of indicators of patient centeredness in the Quality and Outcomes Framework has been mooted as a possible incentive for SDM.^6^ Leaders in the field of SDM are engaged in a series of roundtable discussions, facilitated by the Institute of Medicine, tasked with identifying valid and reliable measures of SDM. Without such measures, accurate assessments of clinical performance will be difficult and the impact of incentives on the implementation of SDM impeded.

## Patient‐reported measures of shared decision making

Scholl *et al*.^7^ reviewed the psychometric properties of SDM measures, 13 of which were PRMs of either SDM process or outcomes.^8^, ^9^, ^10^, ^11^, ^12^, ^13^, ^14^, ^15^, ^16^, ^17^, ^18^, ^19^, ^20^, ^21^ PRMs are often dichotomized into patient‐reported outcome measures (PROMs), a patient report of their health status, or patient‐reported experience measures (PREMs), a patient reported of their healthcare experience. SDM measures tend to fall into the later category. Measures of SDM were both unidimensional,^9^, ^20^, ^21^ and multidimensional,^8^, ^10^, ^17^ with dimensions including, for example, ‘patient satisfaction with decision’, ‘decisional control’ and ‘support provided by clinician when making a decision’. The review found good reliability statistics for included measures; however, the validity of many measures remained undetermined and patient involvement in measure development was unclear. Patient involvement in item conception and formulation is recommended in practice^22^, ^23^, ^24^, ^25^ and essential to the production of health measurement tools that accurately measure constructs of interest.^26^, ^27^ We supplemented the Scholl *et al*. review by assessing the formulation of survey items in detail and found a lack of reported patient involvement in the PRM development process. In this commentary, we discuss these findings in relation to standards in item formulation for PRMs and suggest directions for future scale development with a practical example from the recent development of *Collabo*RATE, a 3‐item PRM of SDM process.^28^, ^29^

### Measuring patient involvement in decisions without patient involvement in measure development

The validity and reliability of PRMs rely on the generation of instructions, items and response options that are understandable to the target audience.^25^, ^26^, ^27^ This is not ‘a trivial task, as no amount of statistical manipulation after the fact can compensate for poorly chosen questions’.^27^ Without investigating patient interpretation and comprehension of the PRM, scores have unknown meaning. For example, misalignment between patients' interpretation of the Control Preferences Scale and intended meaning have been reported.^30^, ^31^ When patients were asked to explain why the care they received led them to indicate on the Modified Control Preferences Scale ‘the doctor and I made the decision together’, few descriptions were aligned with researchers' criteria for genuine SDM. As one patient stated, ‘he [the doctor] made it [the decision] and I agreed’.^32^ This misalignment can lead to the overestimation of levels of SDM in studies that have used this PRM.

Only four of the 13 PRMs reviewed^8^, ^12^, ^17^, ^19^ explicitly state that patients were involved in the conception or development of the measures (Table 1). Two papers made a perfunctory effort to describe patient involvement.^8^, ^19^ The development of the combined outcome measure for risk communication and treatment decision‐making effectiveness (COMRADE) was based on the results from a series of focus groups, designed to identify important outcomes of consultations for consumers of health care.^17^ However, the focus groups were not designed explicitly to inform the creation of a survey, rather the realization that a survey may be needed was a conclusion drawn from the project. Dyadic OPTION,^12^, ^13^ a measure of SDM assessing both clinician and patient perspectives, was the only measure with an independent article describing the detail of item development.^12^ For Dyadic OPTION, three cycles of cognitive interviews (see Section ‘Engaging patients in development using cognitive interviews’ for further details the cognitive interviewing method) were conducted with 27 participants from the general public in the UK. Semantic and grammatically difficult terms were identified, construct mismatch was corrected, and patient preferences for item wording were used to create the final set of 12 items.

**Table 1:** Development characteristics of patient reported measures of shared decision making

Lead author	Measure	Patient involvement in item construction	Decision awareness assumed	Number of decision points evaluated	Sample PRM item
SDM process measures					
Smoliner^8^	Scale on participation in nursing care decisions	Yes – Not described	Yes	Multiple unspecified decisions	‘[I make the decisions myself]’^a^
Kritson^9^	Shared decision making questionnaire (SDM‐Q‐9)	Not reported	Yes	Single specified decision	‘My doctor made clear that a decision needs to be made’
Martin^10^	Facilitation of patient involvement in care scale	Not reported	Yes	Multiple unspecified decisions	‘My doctor gives me all the information that I need to make the decisions that are right for me’
Lerman^11^	Perceived involvement in care scale (PICS)	Not reported	Yes	Multiple unspecified decisions	‘My doctor asked me whether I agree with his/her decisions’
Melbourne^12^, ^13^	Dyadic ‘observing patient involvement in decision making’ (OPTION) Scale	Yes – Cognitive interviews	Yes	Single specified decision	‘The possibility of coming back to the decision was discussed’
SDM outcome measures					
Sainfort^14^	Decision attitude scale	Not reported	Yes	Single unspecified decision	‘My decision is sound’
Stalmeier^15^	Decision evaluation scale	Not reported	Yes	Single unspecified decision	‘This decision is made without me’
O'Connor^16^	Decisional conflict scale	Not reported	Yes	Single unspecified decision	‘This decision is easy for me to make’
Edwards^17^	Combined outcome measure for risk communication and treatment decision‐making effectiveness (COMRADE)	Yes – semi‐structured focus group and interviews	Yes	Single unspecified decision	‘The doctor gave me a chance to be involved in the decisions during the consultation’
Légaré 18	Sure of myself; understand information; risk‐benefit ratio; encouragement (SURE) screening test	Not reported	Yes	Single unspecified decision	‘Do you know the benefits and risks of each option’
Gagnon^19^	The health care empowerment questionnaire (HCEQ)	Yes – interviewed for comprehension	Yes	Multiple unspecified decisions	‘That you and your loved ones decide the need for the health care and services’
Brehaut^20^	Decision regret scale	Not reported	Yes	Single specified decision	‘It was the right decision’
Holmes‐Rovner^21^	Satisfaction with decision scale	Not reported	Yes	Single specified decision	‘The decision I made was the best decision possible for me personally’

a Translated from German to English.

The general lack of patient involvement contrasts with the ethos of patient engagement central to SDM. It is therefore unsurprising that content validity of existing tools has been difficult to demonstrate.^7^, ^33^ It may be that patient involvement in the design of existing PRMs was conducted and not reported, but this possibility also speaks to the lack of emphasis placed on this aspect of measure development. This lack of emphasis is further supported by the limited attention paid to assessing patient involvement in PRM development in current quality appraisal tools for PRMs.^34^, ^35^

### Resulting threats to validity

Two common assumptions underlie existing patient‐reported measures of SDM: (i) an assumption of patient awareness of ‘decision points’ and (ii) an assumption that there is only one decision point in each healthcare consultation (Table 1). Yet, literature would suggest that these assumptions are often not met, threatening the content validity of resulting measurement.

#### Assumption of patient awareness of decision points: ‘The decision? What decision?’

All 13 PRMs of SDM assume that patients are aware that a decision has been made (Table 1), and all but one^18^ include the term ‘decision.’ However, several authors^30^, ^32^, ^36^, ^37^ have noted instances where, to an independent observer, a seemingly obvious decision has occurred but patients are unaware. For example, in a study of women who had undergone a hysterectomy, some women had difficulty identifying when a discrete decision to have a hysterectomy occurred.^30^ Further work by Entwistle^32^ in a general practice population found that patients struggled to identify a decision point because they did not know what constituted a decision. Additionally, Davey *et al*.^31^ report that patients consider some treatment so obvious that there is no decision to be made.

There are many instances where decisions are made implicitly during consultations and may not be recognized by patients. This is especially the case where procedures have become routinized in health care, such as a ‘diagnostic’ performed following the identification of a breast lump.^31^ Also, where patients see no acceptable alternative, they may implicitly assume that no decision‐making opportunity exists; when faced with a decision about paediatric allogeneic blood and marrow transplantation, 81% of parents reported that there was no decision to be made.^37^ In such examples, the use of the terms ‘decision’ or ‘option’ may not be appropriate because patients believe they are ‘agreeing to a plan’ rather than encountering a decision‐making point.^37^

More work is required in defining what actually constitutes a decision.^38^ In a recent study, discordance between patients’ and clinicians’ perceptions of whether a specific decision was made occurred in 30% of consultations. In half of the consultations where discordance was reported, the patient felt a decision was made and the provider did not, with the opposite true in the remaining consultations.

Assuming that patients are always aware of decision points introduces measurement bias, limiting the validity of existing PRMs. Patients cannot accurately answer questions about the processes or outcomes of a decision if they are not aware that a decision has taken place, and though in some instances, where it is known in advance of the consultation that the patient will encounter an explicit decision, the risks associated with this assumption are minimal.^9^, ^20^, ^21^ A solution may be to remove terms such as ‘decision’ from PRMs and use wordings that are better understood by patients (an example is provided in Section ‘Engaging patients in development using cognitive interviews’).

#### An assumption that there is only one decision point: ‘The decision? Which decision?’

Nine of the 13 existing PRMs^8^, ^10^, ^11^, ^19^ require patients to respond in reference to a single decision point. In two of these measures, the decision point is specified in advance by the PRM administrator^20^, ^21^ and, in one, by the patient,^9^ but in seven other measures, it remains unspecified. This approach is at odds with the reality that healthcare consultations involve, on average, seven distinct decision points (3–11 per consultation).^39^, ^40^, ^41^, ^42^ Such decision points can range in complexity, as described by Braddock,^41^ from decisions involving basic levels of complexity such as ordering a laboratory tests, to intermediate complexity, such as making changes to medication, to highly complex decisions, such as discussing the need for a screening test.

Focusing on a single decision point may be appropriate if the objective is to focus on a single specified decision of interest. However, this is less appropriate if the goal of measurement is to assess SDM processes or outcomes globally across a healthcare consultation. Given evidence that variation exists in the level of SDM among different decision points within a consultation,^39^, ^40^ focusing only on one decision carries a risk of over‐ or underestimating the overall level of SDM. Asking patients to complete a survey for each decision they have identified is one way to address this limitation, but it carries significant respondent burden and is difficult to integrate with existing clinical workflows and patients may be unaware that the decisions had taken place. Measures that are broad in scope and assess SDM processes across the consultation – that is, they require patients to provide ‘average’ or global assessments – are another option, and short, generic PRMs have demonstrated validity and reliability in other fields.^43^, ^44^, ^45^ SDM relies on skills that can be taught to clinicians and applied to the whole consultation, rather than an approach to care sanctioned for a limited number of acute, one–off decisions.^46^

As decisions do not always occur as the result of a discrete one–off process during a single consultation,^47^ designing measures that account for prior experiences is a challenge that warrants further detailed exploration.

## Engaging patients in development using cognitive interviews

Existing measures have been designed chiefly for research purposes and without patient input. Future surveys need to be tailored for clinical implementation, namely they need to be short and easy to complete without compromising their ability to discriminate between high and low quality of the SDM process.

Within the field of SDM measurement, there is a need to develop key concepts in a rigorous way, based on literature reviews, expert opinions and patient perspectives. Interviews and focus groups provide a way to examine the concordance between the intended measurement concept and the way patients from the target population understand concepts.^24^, ^25^ Once clearly defined, initial items can then be generated, with patients, to measure the concepts. The inclusion of patients in the conception of items and the identification of constructs that matter most to them is a pertinent, but often overlooked step. It is important that these initial items are open to refinement, with cognitive interviews identified as the recommended method to inform this process.^26^, ^27^ There are two main approaches to cognitive interviewing, think aloud and verbal probing. Verbal probing is the recommended approach for the assessment of item comprehension. It involves the interviewer using a series of pre‐specified questions to ‘probe’ further into the interviewee's responses.^26^ However, cognitive interviews are not without limitations. As a qualitative method, cognitive interviews may generate a sample unrepresentative of the target populations. In addition, the cognitive interview setting is often a different environment from the setting in which the test material, such as surveys, will be administered. Interviewees tend to pay more attention to materials during a cognitive interview than they would in real world administration, leading to unanticipated problems. However, the risk of this can be mitigated if interviewees are not enticed to focus on materials they find boring or confusing.^26^

Table 2 provides an illustrative example of the application of cognitive interviewing to assess comprehension and face validity of *Collabo*RATE, a 3‐item measure of SDM processes which attempts to capture both implicit and explicit decision making over an entire consultation.^28^ We conducted two stages of cognitive interviews and piloted the refined survey with 30 patients. In an attempt to mitigate potential limitations of the cognitive interview method, we purposefully sampled interviewees to ensure an equivalent distribution of age and gender; however, we were unable to do this for education, and the majority of our sample had a university‐level education.

**Table 2:** Cognitive interviews: key features for item development (Adapted from Willis^26^ and Patrick^25^)

Key features	Description	A practical example – the development of *Collabo*RATE
Cognitive focus	Study the cognitive processes that respondents use to answer survey questions, particularly *comprehension* during item development. Detect problems with survey questions	Investigate patient comprehension and refine a series of items that were designed to capture the main dimensions of shared decision making process
Develop items	Draft initial instructions, items and response scales based on criteria for item selection that correspond to the concept under investigation	Drafted three items per dimension of SDM, with the aim of using one well understood item per dimension. Key dimensions for SDM: (i) explain health issue, (ii) elicit patient preferences and (iii) integrate patient preferences
Timing of interviews	Interviews should be conducted between initial drafting and administration of items in the field, to allow pre‐testing and refinement of items	Interviews were conducted after the initial drafting of the items. Items were refined, finalized and piloted with patients for understanding and completion time
Interviewers	Interviewers should be trained in cognitive interview techniques	Standard training in cognitive interview techniques took place over a 2‐hour seminar delivered by an experience qualitative researcher. Pilot cognitive interviews were conducted and recorded. Trainees met again to listen to the interviews, ensuring consistency in the cognitive interview technique
Specialized recruitment	Participants should be selected with particular characteristics of interest, for example in constructing a shared decision‐making measure for women considering breast cancer treatment, women who have had breast cancer may be an appropriate group to recruit	The target population for *Collabo*RATE is the general population of patients attending a consultation with a health professional. Therefore, we purposefully sampled an equal distribution of participants based on gender and age
Use of verbal report procedures	For item development, the use of verbal probing is recommended. For example, ‘what does this term mean to you?’ ‘Can you repeat the question in your own words?’ (further examples of standard probes are available in Willis^26^)	*First impression probes*: Is there anything you find confusing or poorly worded?
		*Assessing words/terms that may be unfamiliar*: What does the term ‘healthcare provider’ mean to you?
		*Understanding of phrasing*: ‘What does the term ‘how much effort’ mean to you?
		*Face validity of the item*: In your own words, what do you think the question is asking?
Sample size	Between 5–15 participants are required per round of interviewing	We recruited 12 participants during stage 1 (initial item review and refinement) and 15 participants during stage 2 (second stage of item review and refinement)
Iterative testing	Following each round of testing, review and modification, the questionnaire is tested in a further round. An item‐tracking matrix should be completed showing each stage of item development. Complete rounds until saturation are reached, where no problems in item comprehension are found	We developed an item matrix (available from Elwyn *et al*. 28) and reached saturation of user issues after two stages of cognitive interview. Saturation of user issues and time to completions were assessed through piloting the final survey with 30 patients following a consultation with a plastic surgeon
Length of interview	Interviews should last no longer that 1 hour	30 min, on average
Settings	Interviews should be conducted in person and in private. With the permission of the participants, recordings should be made and field notes taken during the interview. Notes should be reviewed immediately following the interview	Participants were invited to a private room in the hospital and offered some refreshments during the interview. All participants consented to recordings and interviews were analyzed within 24 h

Interviewees found the term ‘preference’ difficult to comprehend and preferred the phrase ‘what matters most’. The term ‘health problem’ was perceived as negative by interviewees, leading to the use of ‘health issue’. Not only were items refined through the cognitive interview process, item generation also occurred. *Collabo*RATE initially consisted of two items that assessed the core elements of SDM, (i) providing the patient with an explanation of health information and (ii) patient preference elicitation. Yet, a key insight from the first rounds of cognitive interviews led to the inclusion of a third item, preference integration, directly derived from a patient interview. This item assesses the extent to which patient preferences are integrated into a decision. The inclusion of patients as part of the study team may have helped identify this issue earlier in the project. We now include patients in all projects originating from the ‘preference laboratory’, Dartmouth College.

We hope this practical example can act as a roadmap, informing an important facet of the development and assessment of PRMs in SDM.

## Conflict of interests

The authors declare no support from any organization for the submitted work; Prof. Elwyn reports grants and personal fees from the Informed Medical Decision Making Foundation and from Emmi Solutions LLC, outside the submitted work. As two of the copyright holders of *Collabo*RATE, Prof. Elwyn and Dr. Barr wish to declare this intellectual conflict of interest. *Collabo*RATE is also freely available under a Creative Commons License for non‐commercial use‐ CC BY‐NC‐ND *3.0 Unported*. *Collabo*RATE is available under license for commercial organizations, to date no fees have been levied for this.

## Funding

This project was unfunded.
